# Checklist for Reproducibility of Deep Learning in Medical Imaging

**DOI:** 10.1007/s10278-024-01065-2

**Published:** 2024-03-14

**Authors:** Mana Moassefi, Yashbir Singh, Gian Marco Conte, Bardia Khosravi, Pouria Rouzrokh, Sanaz Vahdati, Nabile Safdar, Linda Moy, Felipe Kitamura, Amilcare Gentili, Paras Lakhani, Nina Kottler, Safwan S. Halabi, Joseph H. Yacoub, Yuankai Hou, Khaled Younis, Bradley J. Erickson, Elizabeth Krupinski, Shahriar Faghani

**Affiliations:** 1 Mayo Clinic Artificial Intelligence Laboratory, Department of Radiology, Mayo Clinic, 200 1st St SW, Rochester, MN 55905 USA; 2 Department of Orthopedic Surgery, Orthopedic Surgery Artificial Intelligence Laboratory, Mayo Clinic, Rochester, MN USA; 3 Department of Radiology and Imaging Sciences, Emory Healthcare, Emory University, Atlanta, GA USA; 4 Department of Radiology, NYU Langone Health, New York, NY USA; 5 DasaInova, Dasa, Universidade Federal de São Paulo, São Paulo, Brazil; 6 San Diego VA Health Care System, San Diego, CA USA; 7 Department of Radiology, Thomas Jefferson University Hospital, Philadelphia, PA USA; 8 Radiology Partners Research Institute, El Segundo, CA USA; 9 Department of Medical Imaging, Ann & Robert H. Lurie Children’s Hospital of Chicago, Chicago, IL USA; 10 Department of Radiology, MedStar Georgetown University Hospital, Washington, DC USA; 11 Department of Electrical Engineering and Computer Science, Vanderbilt University, Nashville, TN USA; 12 Philips Research North America, Cambridge, MD USA; 13 Department of Radiology and Imaging Science, Emory University School of Medicine, Atlanta, GA USA

**Keywords:** Deep learning, Reproducibility, Delphi, Checklist

## Abstract

The application of deep learning (DL) in medicine introduces transformative tools with the potential to enhance prognosis, diagnosis, and treatment planning. However, ensuring transparent documentation is essential for researchers to enhance reproducibility and refine techniques. Our study addresses the unique challenges presented by DL in medical imaging by developing a comprehensive checklist using the Delphi method to enhance reproducibility and reliability in this dynamic field. We compiled a preliminary checklist based on a comprehensive review of existing checklists and relevant literature. A panel of 11 experts in medical imaging and DL assessed these items using Likert scales, with two survey rounds to refine responses and gauge consensus. We also employed the content validity ratio with a cutoff of 0.59 to determine item face and content validity. Round 1 included a 27-item questionnaire, with 12 items demonstrating high consensus for face and content validity that were then left out of round 2. Round 2 involved refining the checklist, resulting in an additional 17 items. In the last round, 3 items were deemed non-essential or infeasible, while 2 newly suggested items received unanimous agreement for inclusion, resulting in a final 26-item DL model reporting checklist derived from the Delphi process. The 26-item checklist facilitates the reproducible reporting of DL tools and enables scientists to replicate the study’s results.

## Introduction

The introduction of deep learning (DL) techniques in medical imaging has resulted in a new era of medical tools and applications. These tools, supported by complex algorithms and large datasets, hold the promise of transforming diagnostic accuracy, treatment strategies, and patient outcomes. Yet, as the medical field adopts these advanced technologies, the need for thorough and transparent reporting of the DL tool development process becomes crucial. Transparent documentation allows researchers to explore methodologies, replicate findings, and refine techniques, thereby contributing to the robustness and reliability of DL advancements. As a consequence, this should lead to reproducible reporting. Reproducibility is defined as the capacity to autonomously replicate and achieve consistent outcomes within a study or experiment by employing identical or similar methodologies, data, and conditions.

Numerous studies have shown that reproducing results from DL publications is often challenging. DL studies involve new and different elements not considered in usual reporting rules, making explaining their methodology and results more complex [1]. This situation is acknowledged as part of a broader “replication crisis” recognized across scientific fields. Researchers worry that unverifiable findings bypass checks and become accepted as fact [1–4]. Recent studies in medical imaging related to DL indicate a lack of frequent reporting of crucial study details for reproducibility or the absence of code and data sharing across various papers [1, 5, 6]. To address these reporting challenges, our study uses the Delphi method to develop a practical checklist tailored for scholarly journals, facilitating consistent and transparent reproducible reporting of DL medical tools using Delphi.

The Delphi method, a time-tested approach, has been used in diverse applications across various domains, including health research [7–9]. This method is employed in cases where empirical evidence is lacking or inconsistent and subjective assessments are required. Such circumstances, prevalent in medicine, call for combining expert viewpoints and convictions [10]. Given the widespread application of the Delphi method in healthcare scenarios to ascertain agreement on matters of clinical significance, we have employed a similar approach to formulating a comprehensive guideline for systematically reporting DL medical tools within the medical literature (imaging informatics in particular).

Our research builds upon Delphi principles and adapts them for the digital age through electronic Delphi (e-Delphi) utilization. In contrast to its conventional paper-based counterpart, e-Delphi uses electronic means of communication to conduct the process and takes advantage of technological advancements for collaboration among experts, transcending geographical boundaries and enhancing the efficiency of the consensus-building process. This study aims to improve the reproducibility and the overall reporting quality of DL medical tools, contributing to a more responsible and influential research environment.

## Methodology

Delphi’s conduction process has specific attributes. First, anonymity is achieved via questionnaires, promoting candid responses free from social pressure. Iteration involves multiple questionnaire rounds, enabling opinion adjustments. Controlled feedback occurs between rounds, sharing participants’ opinions and promoting inclusive discussions. Lastly, statistical aggregation at the procedure’s end yields a group judgment expressed as a mean score, utilizing deviations to gauge consensus and offering comprehensive insights beyond simple agreement.

In this e-Delphi study, we sought to develop a comprehensive checklist for the reproducible reporting of DL studies in medical imaging. A panel of experts, distinguished by their significant expertise in medical imaging, artificial intelligence, and deep learning tool development, as well as their prolific contributions to academic research and publications, was chosen. These individuals also bring diverse perspectives from their varied work locations and affiliations with multiple societies, enriching the panel with a broad spectrum of knowledge and experience (Nabile Safdar, Linda Moy, Felipe Kitamura, Amilcare Gentili, Paras Lakhani, Nina Kottler, Safwan S. Halabi, Joseph H. Yacoub, Yuankai Hou, Bradley J. Erickson, and Elizabeth Krupinski). Following established research practices, the Delphi methodology requires a minimum of 10 expert participants [11]. To ensure maximum collaboration, our experts were invited through personalized email invitations, followed by two reminders. Out of the 15 email invitations sent, 11 experts agreed to participate via email correspondence.

Our methodology encompassed a multi-step approach:

(i) Preliminary item generation: to establish a foundation, existing checklists were reviewed by M.M., S.F., B.K., and P.R., along with an in-depth analysis of relevant publications, including Checklist for Artificial Intelligence in Medical Imaging (CLAIM), MINimum Information for Medical AI Reporting (MINIMAR), and Consolidated Standards of Reporting Trials–AI (CONSORT-AI), Standard Protocol Items: Recommendations for Interventional Trials-AI (SPIRIT-AI) [12–14]. We incorporated all relevant checklist items, eliminating those that were either redundant or completely unrelated to the steps of tool development. We retained all pertinent items for our expert panelists to review and make decisions about. This process resulted in creating a primary pool of items to form the basis of our checklist. We identified which checklist items were associated with the various phases of a DL study, from the initial stages of data gathering and curation to the ultimate stages of validation.
(ii) Expert scoring: we designed online scoring forms tailored to each category of items. Our panelists were requested to assess items on a three-point scale: (1) whether the item measures what it intended to measure, (2) whether the item is essential, and (3) whether the item is relevant.
  The first question referred to “face validity” or the extent to which a measurement or assessment instrument appears on its face to measure what it intends. The second one, “essential” or “not essential” and “relevant” or “not relevant,” refers to content validity, as it assesses whether the item is essential and relevant for the study’s purpose and goals. Experts were asked to provide scores using a 5-point Likert scale (1 = strongly disagree, 2 = disagree, 3 = neutral, 4 = agree, and 5 = strongly agree). The experts could provide comments or edits for each item, and at the end of each survey, they could offer general feedback or suggest additional items.
  To facilitate the refinement of initial responses, we conducted two survey rounds, enabling participants to revise their answers. In the second round, experts received the modified questionnaire and additional items derived from the first round outcomes. The scoring process ensured that each item underwent a comprehensive evaluation by the expert panel. The scores provided insights into each item’s perceived meaning validity, necessity, and relevance.
(iii) Data analysis: the scores were analyzed to identify patterns of consensus and divergence among the panelists. This iterative process allowed us to distill the most salient and crucial items from the initial pool, contributing to the formulation of a refined checklist.

For each item, we calculated the content validity ratio (CVR), a linear transformation based on the proportional level of agreement, reflecting the proportion of panel participants who designate an item as essential or relevant.

The formula is

$${\text{CVR}}=\frac{\text{Ne}\mathop{-}\frac{N}{2}}{\frac{N}{2}}$$

where Ne represents the number of panel members rating the item as essential (agree or strongly agree) and *N* denotes the total number of panel members. Given our panel of 11 members, the cutoff value for CVR was 0.59. The cutoff value is context-dependent and varies with the number of panel members. The primary utility of the CVR lies in its ability to assess whether experts’ agreement on the necessity of an item goes beyond what could be attributed to the chance agreement [11, 15]. Throughout each iteration, we computed the mean, standard deviation, and CVR for each checklist to evaluate the essentiality of the DL model reporting items.

## Results

Each round involved sending a maximum of two emails containing the survey link, along with up to two reminders. This resulted in a 100% response rate with no dropouts. Participant demographics were as follows: 82% male (9 out of 11), 100% currently working at academic institutions, 9 practicing radiologists, and 2 PhDs, all with strong backgrounds in informatics. The online survey tool generated pooled results for each round in Excel files [16]. The nominal Likert-scale responses were coded as 1 to 5 for each question. In both rounds, experts responded to 100% of the items for all 3 questions, demonstrating face and content validity.

**Fig. 1:**
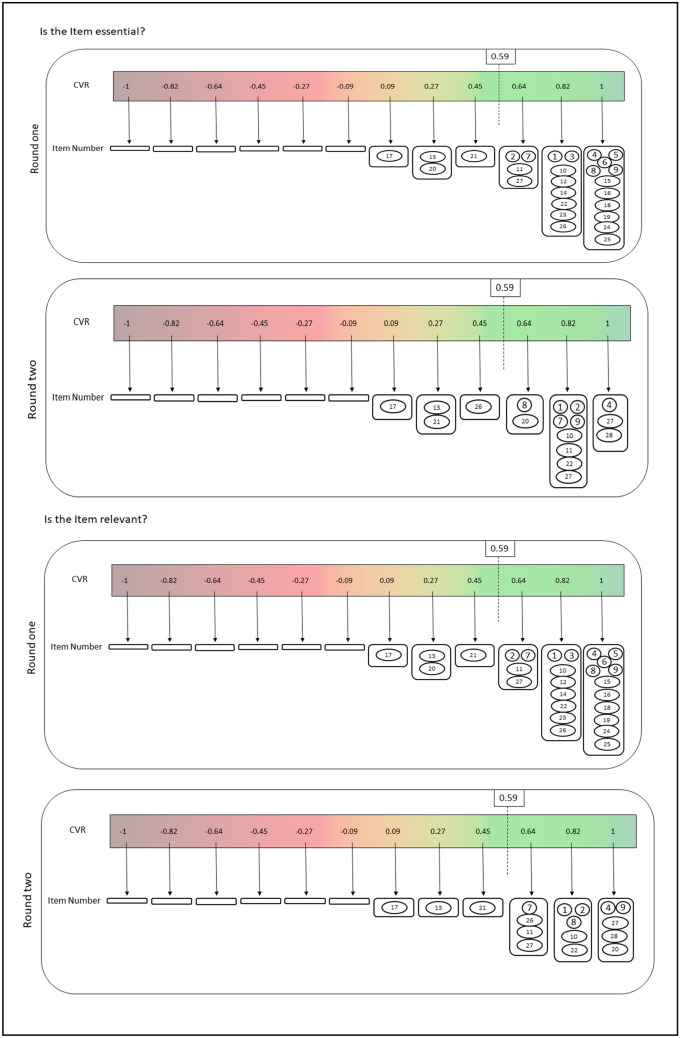
Visualization of the two-round Delphi process outcomes for each checklist item, referencing item numbers from Table 1. The figure delineates the consensus development among 11 expert panelists, with a CVR (content validity ratio) cutoff of 0.59, signifying Delphi consensus

In round one, the 27-item questionnaire was presented to the expert panelists. After the initial round, at least 10 panelists agreed that 12 items had face and content validity. Two items were recommended for inclusion in the questionnaire. Qualitative data were analyzed item by item, with S.F. and M.M. individually assessing comments for similarities. Suggestions and arguments from the first round were integrated into the second round’s items. To foster maximum and optimal collaboration among the panelists in round 2, we eliminated the 12 items with a high level of consensus from the round 1 questionnaire. We retained the remaining items and items that panelists suggested changes to wording. Two new items suggested in round 1 were also incorporated. Consequently, in round 2, the questionnaire had 17 items. In Table 1, we present the results of these two rounds (Fig. 1). The items in italics represent text that was either modified or added during the second round.

**Table 1:** Summary of outcomes from two rounds of Delphi process for each checklist item

	**Item**		**Primary**			**Secondary**		
			**Median**	**IQR**	**CVR**	**Median**	**IQR**	**CVR**
1	Dataset name	Face validity	4	0.5	0.64	4	0.5	0.64
		Validity	4	1	0.82	4	1	0.82
		Feasibility	4	1	0.64	4	0.5	0.82
2	Dataset owner *Dataset owner (how to access to the dataset)*	Face validity	4	1	0.64	4	1	0.64
		Validity	4	0	0.64	4	1	0.82
		Feasibility	4	0.5	0.45	4	1	0.82
3	Time coverage of data	Face validity	4	1	0.82	The item not presented in the second round due to the consensus in the first round		
		Validity	4	0.5	0.82			
		Feasibility	4	0.5	0.82			
4	Dataset size *Dataset size (number of patients, studies, and exams)*	Face validity	5	1	0.82	5	1	1.00
		Validity	5	1	1.00	5	0	1.00
		Feasibility	5	1	1.00	5	0	1.00
5	Inclusion/exclusion criteria	Face validity	5	1	1.00	The item was not presented in the second round due to the consensus in the first round		
		Validity	5	1	1.00			
		Feasibility	5	1	1.00			
6	Data pre-processing steps (data normalization/standardization techniques)	Face validity	4	1	0.82	The item was not presented in the second round due to the consensus in the first round		
		Validity	5	1	1.00			
		Feasibility	5	1	1.00			
7	Software/packages used for pre-processing (including version)	Face validity	4	1	0.82	5	0.5	0.82
		Validity	4	1	0.64	5	1	0.82
		Feasibility	4	1	0.64	5	1	0.64
8	Transformations (augmentation techniques) (qualitative) *Transformations (augmentation techniques) (qualitative) (e.g., rotation, affine transformation, Gaussian noise)*	Face validity	4	2	0.27	4	2.5	0.45
		Validity	5	1	1.00	4	1.5	0.64
		Feasibility	5	1	1.00	4	1	0.82
9	Transformations (augmentation techniques) (quantitative) *Transformations (augmentation techniques) (quantitative) (e.g., degree of rotation, amount of translation, mean and standard deviation of Gaussian noise)*	Face validity	5	1.5	0.45	5	1	0.82
		Validity	5	1	1.00	5	1	0.82
		Feasibility	5	1	1.00	5	1	1.00
10	Grouping criteria *Grouping criteria for data splitting (e.g., whether samples are group by patient or study)*	Face validity	4	1.5	0.09	5	1	0.82
		Validity	4	1	0.82	5	1	0.82
		Feasibility	4	1	0.82	5	1	0.82
11	Stratification criteria	Face validity	4	2	0.27	4	0.5	0.64
		Validity	4	1	0.64	4	1	0.82
		Feasibility	4	1	0.64	4	1	0.64
12	Details of cross-validation, nested cross-validation, or similar techniques	Face validity	4	1	0.82	The item not presented in the second round due to the consensus in the first round		
		Validity	4	1	0.82			
		Feasibility	4	1	0.82			
13	Imaging id assigned to each split set (if data is public) or seed number used for splitting (if data is private)	Face validity	4	1	0.09	4	1	0.45
		Validity	4	1.5	0.27	4	1	0.27
		Feasibility	4	1.5	0.27	4	1	0.27
14	The source code for the architecture(s) used [alternatively: denoting the package they used and the version of that]	Face validity	4	1	0.82	The item was not presented in the second round due to the consensus in the first round		
		Validity	4	1	0.82			
		Feasibility	4	1	0.82			
15	Hyperparameters for dynamic components of the model’s architecture (batch norm layers, dropout probability, etc.) [if used something other than the default value]	Face validity	4	0.5	0.64	The item was not presented in the second round due to the consensus in the first round		
		Validity	4	1	1.00			
		Feasibility	4	1	1.00			
16	Usage of pre-trained weights, source of pre-training weights (if weights are pre-trained externally), protocol of pre-training (if weights are pre-trained internally)	Face validity	4	1	1.00	The item was not presented in the second round due to the consensus in the first round		
		Validity	4	1	1.00			
		Feasibility	4	1	1.00			
17	Random initialization algorithm and specification	Face validity	3	1.5	− 0.09	4	1.5	0.27
		Validity	4	2	0.09	4	1.5	0.09
		Feasibility	4	2	0.09	4	1	0.09
18	Training procedure: loss function(s) and their hyperparameter	Face validity	4	1	0.82	The item was not presented in the second round due to the consensus in the first round		
		Validity	4	1	1.00			
		Feasibility	4	1	1.00			
19	Optimizer(s), learning rate, EMA, and their hyperparameter	Face validity	4	1	0.82	The item was not presented in the second round due to the consensus in the first round		
		Validity	4	1	1.00			
		Feasibility	4	1	1.00			
20	Learning rate scheduler(s) and their hyperparameter (if any) *Learning rate scheduler(s) and their hyperparameter (how learning rate changes, if applicable)*	Face validity	4	1.5	0.27	4	1	0.82
		Validity	4	1.5	0.27	4	1	0.64
		Feasibility	4	1.5	0.27	4	1	1.00
21	Criteria for early stopping (if any)	Face validity	4	1.5	0.45	4	1	0.64
		Validity	4	1.5	0.45	4	1.5	0.27
		Feasibility	4	1.5	0.45	4	1	0.45
22	Software/packages used for training *Software/packages used for training and statistical analysis (including version)*	Face validity	4	1	0.82	5	0.5	1.00
		Validity	4	1	0.82	5	0.5	0.82
		Feasibility	4	1	0.82	5	0.5	0.82
23	Number of epochs	Face validity	4	1	0.82	The item was not presented in the second round due to the consensus in the first round		
		Validity	4	1	0.82			
		Feasibility	4	1	0.82			
24	Inference procedure: data pre-processing steps (if different from training)	Face validity	4	1	0.82	The item was not presented in the second round due to the consensus in the first round		
		Validity	4	1	1.00			
		Feasibility	4	1	1.00			
25	Data post-processing steps (if any)	Face validity	4	1	0.82	The item was not presented in the second round due to the consensus in the first round		
		Validity	4	1	1.00			
		Feasibility	4	1	1.00			
26	Whether the difference between sets (or folds) has been investigated	Face validity	4	1.5	0.90	4	1	0.64
		Validity	4	1	0.82	4	1.5	0.45
		Feasibility	4	1.5	0.82	4	1	0.64
27	Metrics, statistical tests are done, evaluation results, and reporting CI for each metric	Face validity	4	1.5	0.45	4	1	0.64
		Validity	4	1	0.64	4	1	0.82
		Feasibility	4	1	0.64	4	1	0.64
28	*Definition of ground truth/gold standard*	Face validity	The item was suggested by panelists in the first round			5	0	1.00
		Validity				5	0	1.00
		Feasibility				5	0	1.00
29	*Performance of subgroup analysis (e.g., age, sex, disease grade)*	Face validity	The item was suggested by panelists in the first round			5	0.5	1.00
		Validity				5	0.5	1.00
		Feasibility				5	0.5	1.00

Items in italics indicate modifications or additions made in the second round. Key statistical measures include median, IQR (interquartile range), and CVR (content validity ratio), providing comprehensive insights into the consensus and variability among responses

In the final round of the Delphi process, panelists agreed that three items were neither essential nor relevant: (1) imaging ID assigned to each split set (if data is public) or seed number used for splitting (if data is private), (2) random initialization algorithm and specification, and (3) criteria for early stopping (if any). The two items suggested by panelists (definition of ground truth/gold standard and performance of subgroup analysis (e.g., age, sex, disease grade)) were strongly agreed upon by all panelists to be essential and relevant for inclusion in the questionnaire. The final checklist, consisting of 26 items, was derived from this Delphi study (Table 2).

**Table 2:** Finalized checklist for reproducible reporting of deep learning studies with 26 items

**Item**	**Check**	**Item**	**Check**
1. Dataset name		2. Dataset owner (how to access the dataset)	
3. Time coverage of data		4. Dataset size (number of patients, studies, and exams)	
5. Inclusion/exclusion criteria		6. Data pre-processing steps (data normalization/standardization techniques)	
7. Software/packages used for pre-processing (including version)		8. Transformations (augmentation techniques) (qualitative) (e.g., rotation, affine transformation, Gaussian noise)	
9. Transformations (augmentation techniques) (quantitative) (e.g., degree of rotation, amount of translation, the mean and standard deviation of Gaussian noise)		10. Grouping criteria for data splitting (e.g., whether samples are grouped by patient or study)	
11. Stratification criteria		12. Details of cross-validation, nested cross-validation, or similar techniques	
13. The source code for the architecture(s) used [alternatively: denoting the package they used and their version]		14. Hyperparameters for dynamic components of the model’s architecture (batch norm layers, dropout probability, etc.) [if used something other than the default value]	
15. Usage of pre-trained weights, source of pre-training weights (if weights are pre-trained externally) and pre-training protocol (if weights are pre-trained internally)		16. Training procedure: loss function(s) and their hyperparameter	
17. Optimizer(s), learning rate, EMA, and their hyperparameter		18. Learning rate scheduler(s) and their hyperparameter (how learning rate changes, if applicable)	
19. Software/packages used for training and statistical analysis (including version)		20. Number of epochs	
21. Inference procedure: data pre-processing steps (if different from training)		22. Data post-processing steps (if any)	
23. Whether the difference between sets (or folds) has been investigated		24. Metrics, statistical tests are done, evaluation results, and reporting CI for each metric	
25. Definition of ground truth/gold standard		26. Performance of subgroup analysis (e.g., age, sex, disease grade)	

## Discussion

In this study, we employed the Delphi methodology to develop a comprehensive checklist for the reproducible reporting of DL studies in the field of medical imaging. The result was a 26-item checklist with high face and content validity. The most optimal and reproducible approach to report on developing a deep learning tool involves sharing both the code and the accompanying data. However, we acknowledge that practical constraints may sometimes make this unfeasible. In such cases, an emerging alternative is to share pseudocode, which provides a higher-level, human-readable representation of the algorithm’s logic. The reporting tool we have introduced is particularly designed for scenarios where sharing code and data is not feasible, offering an effective solution for maintaining transparency and facilitating reproducibility in these situations.

When comparing our checklist to the existing CLAIM checklist, which comprises 42 items, several notable distinctions emerge. The CLAIM checklist is primarily structured around the various sections of a scientific manuscript, emphasizing what should be included in the paper. In contrast, our checklist is tailored explicitly for reporting DL tools in a reproducible manner and does not delve into the broader details of manuscript composition. Elements such as funding sources, title selection, introductions, and study limitations, while relevant to scientific publications, are not imperative for the reproducibility of a DL tool. Published in a journal related to radiology, CLAIM’s scope extends beyond radiology and can be used in the broader field of medical imaging publications [17].

Within the CLAIM methodology and results section, there are commonalities between our checklist and the CLAIM checklist, but distinctions also exist. Both checklists recognize the significance of detailing the software and packages used at each process step. However, our checklist extends its focus by incorporating additional items, such as the reporting of the optimizer, learning rate, batch norm layers, and dropout probability. These finer-grained details are essential for reproducing results in the context of DL model development. Conversely, some aspects emphasized in CLAIM, such as the rationale behind ground truth development, though valuable in certain contexts, have been summarized in one item in our checklist to simply encompass the definition of ground truth, eliminating the need for extensive explanation. We believe providing a detailed rationale, while important, will not significantly impact the steps for tool development.

Compared to the MINIMAR checklist, which is not specifically tailored for DL tools and encompasses machine learning tools as well, notable differences emerge in its scope and focus. MINIMAR includes items like feature selection and identifying the intended users, such as clinicians or insurance companies. However, we found these elements less relevant when it comes to reproducing DL tools. While our checklist shares certain commonalities with MINIMAR, we went into more detail on individual items because reproducing DL tools often requires in-depth information. A prominent example of this is our breakdown of the “Model evaluation Optimization” item in MINIMAR, which we expanded to encompass a comprehensive exploration of various evaluation methods and optimization techniques. This approach reflects our commitment to gathering detailed information for rigorously reproducing DL tools [13].

Compared to tools like CONSORT-AI or SPIRIT-AI (short for Consolidated Standards of Reporting Trials–AI and Standard Protocol Items: Recommendations for Interventional Trials-AI, respectively), it is important to note that these checklists are specifically tailored for trials, encompassing aspects such as blinding, trial design, and interventions [17]. These checklists excel in addressing the unique requirements of AI in trials. While we acknowledge that trials involve elements beyond code replication, for the AI component—if it played a crucial role in analysis and predictive model development—comprehensive details in our checklist are essential for achieving consistent and reproducible results [14].

One important consideration when creating a checklist is to avoid making it overly lengthy. The goal is to ensure that users can realistically address all the required items. While there are numerous small details in the world of DL model tool development, it is not feasible to request developers to provide every single one. Therefore, we aimed for a balance between reporting what is necessary for reproducibility and being comprehensive in covering all the essential aspects. We hope that developers will continue to report any new techniques that may be incorporated into model development within the inclusive sections.

In conclusion, this e-Delphi study successfully developed a robust and contextually relevant checklist for the reproducible reporting of DL studies in medical imaging. The engagement of an expert panel, the rigorous content validation process, and the use of the CVR were pivotal in shaping this valuable tool. We hope this checklist will serve as a guiding framework, fostering transparency and methodological rigor in DL research and ultimately contributing to improved healthcare outcomes. Future studies will assess the rate of adoption in the medical imaging informatics literature and, if possible, the degree of compliance.

## Abbreviations

DL: Deep learning
CVR: Content validity ratio
