# Correction: Checklist for Reproducibility of Deep Learning in Medical Imaging

**DOI:** 10.1007/s10278-024-01295-4

**Published:** 2024-10-22

**Authors:** Mana Moassefi, Yashbir Singh, Gian Marco Conte, Bardia Khosravi, Pouria Rouzrokh, Sanaz Vahdati, Nabile Safdar, Linda Moy, Felipe Kitamura, Amilcare Gentili, Paras Lakhani, Nina Kottler, Safwan S. Halabi, Joseph H. Yacoub, Yuankai Hou, Khaled Younis, Bradley J. Erickson, Elizabeth Krupinski, Shahriar Faghani

**Affiliations:** 1 Mayo Clinic Artificial Intelligence Laboratory, Department of Radiology, Mayo Clinic, 200 1st St SW, Rochester, MN 55905 USA; 2 Department of Orthopedic Surgery, Orthopedic Surgery Artificial Intelligence Laboratory, Mayo Clinic, Rochester, MN USA; 3 Department of Radiology and Imaging Sciences, Emory Healthcare, Emory University, Atlanta, GA USA; 4 Department of Radiology, NYU Langone Health, New York, NY USA; 5 DasaInova, Dasa, Universidade Federal de São Paulo, São Paulo, Brazil; 6 San Diego VA Health Care System, San Diego, CA USA; 7 Department of Radiology, Thomas Jefferson University Hospital, Philadelphia, PA USA; 8 Radiology Partners Research Institute, El Segundo, CA USA; 9 Department of Medical Imaging, Ann & Robert H. Lurie Children’s Hospital of Chicago, Chicago, IL USA; 10 Department of Radiology, MedStar Georgetown University Hospital, Washington, DC USA; 11 Department of Electrical Engineering and Computer Science, Vanderbilt University, Nashville, TN USA; 12 Philips Research North America, Cambridge, MD USA; 13 Department of Radiology and Imaging Science, Emory University School of Medicine, Atlanta, GA USA

**Correction: Journal of Imaging Informatics in Medicine (2024) 37:1664-1673**

10.1007/s10278-024-01065-2

This paper has been republished with open access.

